# Lattice-charge imbalance and redox catalysis over perovskite-type ferrite- and manganite-based mixed oxides as studied by XRD, FTIR, UV–Vis DRS, and XPS

**DOI:** 10.1038/s41598-023-34065-3

**Published:** 2023-05-08

**Authors:** Gamal A. H. Mekhemer, Hagar A. A. Mohamed, Ali Bumajdad, Mohamed I. Zaki

**Affiliations:** 1 Chemistry Department, Faculty of Science, Minia University, El-Minia, 61519 Egypt; 2 Chemistry Department, Faculty of Science, Kuwait University, P.O. Box 5969, 13060 Safat, Kuwait

**Keywords:** Catalysis, Surface chemistry

## Abstract

In the present investigation, two sets of pure and substituted ferrite- and manganite-based mixed oxides were prepared within the stoichiometric formula$A_{1 - x} A^{\prime}_{x} B_{1 - x} B^{\prime}_{x} O_{3}$, where *A* = Bi or La, *A*^′^ = Sr, *B* = Fe or Mn, *B′* = Co, *x* = 0 or 0.2, by calcination at 700 °C (for 1 h) of corresponding metal citrate xerogels. Materials thus obtained were examined for bulk and surface characteristics using X-ray diffractometry, ex situ Fourier transform infrared spectroscopy, UV–Vis diffuse reflectance spectroscopy, X-ray photoelectron spectroscopy, and N_2_ sorptiometry. Their redox catalytic activity was evaluated towards a 2-propanol dehydrogenation reaction in the gas phase by employing in situ Fourier transform infrared spectroscopy. The results obtained could help reveal that (1) the presence of Bi (versus La) and Mn (versus Fe) facilitated the formation of polymeric crystalline phases assuming lattice-charge imbalance (due to excess positive charge), (2) the surface exposure of the excess positive charge was manifested in the generation of Mn sites having various oxidation states ≥ 3+, (3) the consequent development of visible light absorptions at 498–555 nm suggested occurrence of electron double-exchange facilitated by the formation of Mn^n+^–O^2−^–Mn^(n+1)+^ Zener-type linkages, and (4) the exposure of such linkages at the surface warrants the establishment of the electron-mobile environment necessitated by the redox catalytic activity. Moreover, the relationship between the alcohol dehydrogenation activity and the magnitude of the lattice-charge imbalance (i.e., the net excess positive charge) of the catalysts was highlighted.

## Introduction

Complex redox processes (e.g., methane steam reforming^1^ and automobile exhaust gas catalytic conversion^2^) are of particular industrial and environmental importance^2,3^. Enhancing such complex processes, however, requires polyfunctional catalysts^4^. Catalytic polyfunctionality has frequently been sought on surfaces of mixed metal oxides, where a variety of interacting redox-active sites (such as vacancies, coupled oxidation states, coordinatively unsaturated sites, etc.) are made available^5^.

Optimal mixing of metal oxides (e.g., *AO*_*x*_ and *BO*_*x*_) is achieved chemically at the atomic level. This is best accomplished in spinel- (*AB*_*2*_*O*_*4*_) or perovskite-type (*ABO*_*3*_) solid lattices^5–7^. The latter (perovskite) type is advantageous to the former (spinel) type by (1) its ability to accommodate ca. 90% of the metallic elements of the periodic table^6^, and (2) its tolerance to much more partial substitutions of cations in positions *A* and *B*, giving rise to a wide range of substituted compounds assuming the general formula $A_{1 - x} A^{\prime}_{x} B_{1 - x} B^{\prime}_{x} O_{3}$^6,7^. Therefore, various methods have been devised to obtain perovskite-type pure and substituted mixed oxides of varied particle sizes and shapes and physical and chemical properties^6,8,9^ in response to different specific demands of the wide range of applications they enjoy^6,10^. For instance, the weak total magnetic moment in the magnetically ordered state of bismuth orthoferrite-based materials was turned into excellent magnetodielectric properties when other classes of functional iron oxide materials were prepared^10,11^.

The perovskite-type structure assumes a perfect (or distorted) cubic lattice made up of 8 corner-sharing octahedra (*BO*_*6*_) in which the *B* atoms are slightly off-centered, and 12-coordinated *A* atoms dwell in the middle^6,12^. The *B* atoms are transition metals, whereas the *A* atoms are larger lanthanides and alkaline earths^6,7,12^. Electron diffraction and transmission electron microscopy lattice images^13^ have evidenced long-range charge-ordering in perovskite-type, substituted metal oxides (Bi_1−x_Sr_x_MnO_3_ and La_1−x_Ca_x_MnO_3_), including transition metal ions (Mn) assuming different oxidation states (Mn(III) and Mn(IV)). The existence of the transition *B* metal atoms in two different oxidation states (*B*^*n*±1^) eventually results in charge-imbalanced lattices dwelling lined-up *B* metal octahedra (*BO*_*6*_) of symmetric and asymmetric (Jahn–Teller distorted) configurations^13^. Through-oxygen linkages between these differently charged *B* atoms, such as *B*^*n*^–O^2−^–*B*^*n*+1^, have been reported^14^ to facilitate electron double-exchange interactions (Zener mechanism^15^) between the metal atoms. Consequently, the surface electron mobility is enhanced, and hence, the surface redox catalytic activity^16^. Furthermore, the deviation of the concentration of the original cations from a given value can change the charge state of the transition metal cations, considerably changing the material's magnetic and electrical parameters^17^. The oxygen excess and deficit can increase and decrease the oxidation state of 3d-metals. Consequently, the total magnetic moment, Curie point, electrical conductivity, bandgap, and intensity of exchange interactions (electron mobility) are changed^18^.

The present investigation was designed to evaluate the redox catalytic activity of the title perovskite-type mixed oxides, in an attempt to determine the extent of its dependence on the catalysts’ crystalline phase composition, with special emphasis on the lattice-charge imbalance. To accomplish this objective, pure and substituted perovskite-type $A_{1 - x} A^{\prime}_{x} B_{1 - x} B^{\prime}_{x} O_{3}$ (where *A* = Bi or La, *B* = Fe or Mn, *A′* = Sr, *B′* = Co, and *x* = 0 or 0.2) mixed oxides were prepared and subjected to (1) X-ray powder diffractometry and ex-situ Fourier-transform infrared spectroscopy for identification of structures formed in the bulk crystalline and noncrystalline domains, (2) UV–Vis diffuse reflectance spectroscopy for detecting compositions facilitating electron availability and mobility, (3) X-ray photoelectron spectroscopy for probing the surface elemental composition and oxidation states, and (4) N_2_ sorptiometry for specific surface area determination. Furthermore, in-situ FT-IR spectroscopy was employed to observe chemical changes conceded by 2-propanol gas phase molecules, whereby the alcohol dehydrogenation activity of the test catalysts was evaluated. It is worth highlighting that the novelty of the present work lies essentially in the attempt to use a simple molecular stoichiometry calculation, as applied to the observed phase composition of the material bulk, to predict the surface redox catalytic activity of the test mixed oxides.

## Methods

### Catalyst preparation

The test catalysts were pure (*x* = 0) or substituted (*x* = 0.2) mixed oxides derived in the perovskite-type stoichiometric range $A_{1 - x} A^{\prime}_{x} B_{1 - x} B^{\prime}_{x} O_{3}$ (where *A* = Bi(III) or La(III), *B* = Fe(III) or Mn(III)*, A′* = Sr(II), and *B′* = Co(II)). They were obtained by calcination at 700 °C of a carbonized xerogel of the metal citrate. The xerogel was synthesized by applying a modified version^19^ of the method devised by Baythoun and Sale^20^. Accordingly, appropriate amounts of 98%-pure LABO Chemie (India) products of the *A*_*1−x*_*/A′*_*x*_ and *B*_*1−x*_*/B′*_*x*_ nitrate compounds and citric acid monohydrate to furnish a 1:1:1 molar ratio were dissolved in 30 mL of distilled water plus 5 mL of 33%-HNO_3_ acid (Sigma-Aldrich). After magnetic stirring at room temperature (RT) untill complete dissolution, the temperature was increased slowly up to 80 °C and maintained till complete dryness (*ca.* 48 h). Subsequently, the resulting metal citrate xerogels were crushed into a fine powder, placed into porcelain crucibles, and subjected to a carbonization process via two successive heating cycles in still air between RT and 400 °C (at 20 °C/min) in a muffle furnace. The carbonized products were fine powdered and then subjected to calcination (10 °C/min, in a still atmosphere of air) at 700 °C for 1 h. For control purposes, simple oxides of the metals used (i.e., *AO*s and *BO*s) were similarly prepared and calcined. Untill further use, the calcination products were kept dry over CaCl_2_.

For simplicity, the calcination products of the pure and substituted mixed oxides are denoted below as, for example, BFO, (B,L)FO, (L,S)MO or L(M,C)O, where B=Bi, L=La, F=Fe, M=Mn, C=Co, and S=Sr. Hence, for instance, BFO denotes pure BiFeO_x_, and (B,S)FO denotes Sr-substituted (Bi_0.8_Sr_0.2_)FeO_x_. Analogously, the control simple oxides are designated FO, MO, LO, and BO.

### Catalyst characterization

The catalysts' bulk crystalline phase composition, purity, and electronic properties were determined by X-ray powder diffractometry (XRD), ex situ Fourier transform infrared spectroscopy (ex situ IR), and UV–Vis diffuse reflectance spectroscopy (DRS), respectively. The surface chemical composition and accessibility were determined by X-ray photoelectron spectroscopy (XPS) and BET analysis of N_2_ sorptiometry data (S_BET_), respectively. XRD diffractograms were acquired (at 5–100°, 2°/min, 40 kV, 30 mA, and λ = 0.15418 nm) employing JSX-60PA Jeol diffractometer (Japan) and installed JCPDS files^21^ for crystalline phase identification. Ex-situ IR spectra were taken (at 4000–400/cm and a resolution of 4/cm) from lightly loaded disks of KBr-supported test samples on a Genesis-II Mattson Fourier transform infrared spectrophotometer (USA). UV–Vis DRS spectra were taken (at 200–800 nm) from compacted disks of test samples using a Shimadzu 2100 double-beam spectrometer (Japan) equipped with DR attachment and reference material of BaSO_4_ (Shimadzu Corp.). XPS spectra were recorded by using a Thermo ESCALAB-250Xi spectrometer (Thermo Scientific, UK). The radiation source was monochromatic with an AlKα radiation power 1486.6 eV (anode HT of 15 kV, 10 mA) and spot size 850 μm. The vacuum in the analysis chamber was better than 1 × 10^–9^ mbar, and the binding energy (BE/eV) determination was based on the carbon contamination C1s at 284.6 eV with an experimental error of ± 0.2 eV. The specific surface area (S_BET_/m^2^/g) was determined by BET analysis^22^ of N_2_ physisorption data measured (at − 195 °C) after test sample degassing (at 200 °C for 1 h) employing Nova 2200 Quantachrome gas sorptiometer (USA).

### Catalytic activity measurements

The redox activity of the test catalysts was evaluated towards the dehydrogenation of 2-propanol (2-PrOH) gas-phase molecules by using in-situ FT-IR spectroscopy and a homemade all-Pyrex glass IR-reactor/cell^23^ equipped with CaF_2_ windows and hocked to a homemade all-Pyrex Gas/Vac line. Following a 30-min evacuation at RT (to 10^–5^ Torr) of an accurately weighed portion (80–100 mg) of the catalyst particles placed in a specially designed Pyrex-glass bed located inside the cell, the cell background spectrum was taken (averaged 20 scans, 4000–400/cm, 4/cm resolution, using the Matteson II spectrophotometer). Then, an accurately measured portion (27–30 Torr) of the alcohol vapor was admitted into the cell by volume expansion of the vapor of a daerated source liquid of the alcohol (Specpure Merck product). After a 5-min contact time with the catalyst at RT or 250 °C, a spectrum was taken from the gas phase. With the help of the installed WinFirst Lit v1.02 software, absorption subtraction of the cell background spectrum yielded a difference spectrum revealing the chemical changes conceded by the 2-PrOH gas phase composition.

For quantitative analysis, the integrated area of diagnostic absorption peaks of the alcohol (νOH at 3644/cm) and its dehydrogenation product (acetone, νC=O at 1740/cm) was measured and used (versus preconstructed area-pressure calibration curves) to determine the partial pressure of each. These values were used to calculate the alcohol conversion% (%Conv) and the intrinsic turn-over-number (TON) as follows:

$$ \% {\text{Conv }}\left( {{\text{per g}} - {\text{Cat}}} \right) \, = \, \left[ {\left( {{\text{P}}_{{\text{i}}} - {\text{P}}_{{\text{T}}} /{\text{P}}_{{\text{i}}} } \right)} \right] \, \times { 1}00 $$

TON = Number of alcohol molecules converted per unit time per unit specific surface area, i.e. 2-PrOH molecules converted/min m^2^; where, P_i_ = the initial alcohol pressure (in Torr) and P_T_ = the alcohol pressure (in Torr) after a 5-min contact with the catalyst at the reaction temperature (T).

## Results and discussion

### Catalyst characteristics

The obtained XRD diffractograms are compared for the simple oxides and the ferrite- and manganite-based pure and substituted mixed oxides in Supplementary Figs. S1–S3(available online), respectively. The composition and abundance of the crystalline phases thereby identified are presented in Table 1. Accordingly, it is shown that (1) neither of the pure and substituted mixed oxides examined allows for the crystallization of segregated simple oxides, and (2) neither of the substituted manganite-based mixed oxides forms ternary-metal phases (i.e., including the substituent Sr or Co ions). Bi (versus La) is shown to facilitate the formation of polymeric crystalline phases more effectively in manganite-based pure and substituted mixed oxides (e.g., o-Bi_2_Mn_4_O_10_ and c-Bi_12_MnO_20_ both in BMO and B(M,C)O) (supplementary Fig. S3). Irrespective of the counterion (Bi or La), the presence of Mn (versus Fe) facilitates the formation of mixed oxides organized in charge-imbalanced lattices. The lattice-charge imbalance (LCI, Table 1), as realized and calculated from the formal molecular composition of the crystalline phases identified, is shown to be due to excess positive charge, whose value is dependent on the elemental constitution of the phase composition. Hence, the excess positive charge may account for the presence of metal ions in > 3+ oxidation state(s).The obtained ex situ IR spectra are shown (Supplementary Figs. S4 and S5) to display strong absorption bands due to ν(M–O)_*L*_ lattice vibrations relevant to the XRD-identified crystalline phases (Table 1) in the low-frequency region (< 700/cm)^24^. Furthermore, weak-to-very weak bands due to νOH (at 3430/cm), νCH (2880/cm), δOH (1630/cm), νNO_3_^–^ (1380/cm), and νCO_3_^2−^ (1460 and 1060/cm)^25^ are also observed. Except for the νCO_3_^2-^ bands (1460 and 1060/cm), which are only observed in the spectra of the La-containing mixed oxides, the rest of these weak bands are found in all of the spectra obtained and can, therefore, be attributed to minority surface species either inherited from the preparation course (νNO_3_^−^) or due to adsorptive interactions with the ambient atmosphere (ν/δOH and νCH). The emergence of the νCO_3_^2−^ bands may be ascribed to the La-improved material basicity (*cf.* the IR spectrum exhibited for the simple La-oxide (LO, Supplementary Figs. S4, and S5). Hence, the obvious weakening of the carbonate absorption bands on the substituted modifications of LFO (i.e., L(F,C)O) and LMO (i.e., (L,S)MO) may imply that the substituents render the La-content either less accessible or less capable of chemisorbing ambient CO_2_ molecules.

**Table 1:** Bulk crystalline phase composition and *d–d* electronic interactivity as determined for the test catalysts and the control simple oxides by XRD and UV–Vis DRS spectra.

Catalyst	Crystalline phase			λ_*d–d*_^d^ (nm)
	Composition^a^	Abundance^b^	LCI^c^ /+ve	
BFO	r-BiFeO_3_	*j*	0.0	495(S),650(W)
	o-Bi_2_Fe_4_O_9_	*m*	0.0	
LFO	c-LaFeO_3_	*j*	0.0	455(S),523(M),685(VW)
	o-LaFeO_3_	*m*	0.0	
(B,S)FO	c-Sr_0.6_Bi_0.4_FeO_2.7_	*j*	0.0	455(bM),555(bM),650(bM)
	c-Sr_0.6_Bi_0.4_FeO_3_	*m*	0.6	
L(F,C)O	c-LaCo_0.4_Fe_0.6_O_3_	*s*	0.0	455(M),495(M),685(W)
BMO	c-Bi_12_MnO_20_	*j*	1.0	453(S),498(S),555(M)
	o-Bi_2_Mn_4_O_10_	*m*	0.5	
LMO	c-LaMnO_3_	*m*	0.0	453(bS),498(S),555(S),626(M)
	o-LaMnO_3.15_	*j*	0.3	
(L,S)MO	o-LaMnO_3.15_	*s*	0.3	453(S),498(S),555(bS),692(bS)
B(M,C)O	o-Bi_2_Mn_4_O_10_	*j*	0.5	453(S),498(S),555(bS),626(bS), 692(bS)
	c- Bi_12_MnO_20_	*m*	1.0	
Simple oxide				
FO	r-Fe_2_O_3_	*s*	N.A.^e^	490(bW)
MO	o-Mn_2_O_3_	*s*	N.A	510 (bS)
BO	m-Bi_2_O_3_	*s*	N.A	–
LO	h-La_2_O_3_	*j*	N.A	–
	m-La_2_O_3_	*m*		
	m-La_2_O_2_CO_3_	*m*		

^a^r, rhombohedral; o, orthorhombic; h, hexagonal; c, cubic; m, monoclinic. ^b^*s*, sole; *j*, major; *m*, minor. ^c^The formal lattice-charge imbalance value (LCI) was calculated considering (1) the molecular chemical composition of the material XRD-identified phases, and (2) the formal oxidation state of the constituting elements (Mn = Bi = La = 3+, Sr = Co = 2+, and O = 2−). ^d^Underlined wavelengths are those suggested to relate to excitations in the electron double-exchange Zener phases^11^; S, strong; W, weak; VW, very weak; M, medium; b, broad. ^e^N.A., not applicable.

In Supplementary Figs. S6 and S7, UV–Vis DRS spectra obtained for the ferrite- and manganite-based mixed oxide catalysts are compared. According to AlSalka et al.^12^, the full wavelength range scanned (200–800 nm) may be classified into the following excitation ranges (I–III): (I) ligand–metal charge transfer transitions (LMCT) at 235–293 nm, (II) ligand field transitions (LFT) at 313–390 and 626–692 nm, and (III) electron double-exchange transitions (EET) at 453–555 nm. It is obvious that the LMCT are high-energy transitions and, therefore, occur in the UV region (≤ 300 nm), whereas the other transition types are of lower energy and, thus, occur in the visible region (> 300 nm). The absorption maxima resolved in the EET and LFT regions are reviewed in Table 1. Supplementary Figs. S6 and S7 show the ferrite-based mixed oxides enjoy stronger and better resolved LMCT and LFT absorptions in the high-energy region, whereas the manganite-based mixed oxides are more distinct by stronger, extended EET and LFT absorptions in the low-energy region. Table 1 reveals that wherever a nonzero LCI value (i.e., excess positive charge) is encountered, a couple of EET-absorption maxima are resolved at 498 and 555 nm. Ellison and Sing^26^ obtained an analogous couple of EET-absorption maxima (at 420–430 and 580–590 nm) for calcined chromia catalysts exposing Cr^≥3+^ sites. In a subsequent publication^27^, the same authors adopted the double-exchange mechanism of Zener^28^, which he implemented to explain ferromagnetism in mixed valency manganites with perovskite structures (e.g., the series (La_x_Ca_1−x_)(Mn_x_^3+^Mn_1−x_^4+^)O_3_). Accordingly, Ellison and Sing^27^ associated the observed couple of EET-maxima to the exposure on the test calcined chromia catalysts of Cr^6+^–O^2−^–Cr^3+^ linkages sustained by electron exchange forces. Hasan et al.^16^ and Nohman et al.^29^ reported that the establishment of such M^n+^–O^2−^–M^(n+1)+^ double-exchange species at surfaces warrants the availability of not only a mobile-electron environment but also strong coordination sites, and both availabilities are necessary for the optimization of surface redox catalysis.

Table 2 sets out the determined specific surface area (S_BET_) for pure and substituted ferrite- and manganite-based mixed oxides. It is evident from the results that the pure mixed oxides (BFO, LFO, and BMO) typically assume low surface areas (≤ 10 m^2^/g)^6^, except for LMO, which is found to assume 16 m^2^/g. In contrast, the substituted mixed oxides ((B,S)FO, L(F,C)O, and (L,S)MO) exhibited higher surface areas (20–21 m^2^/g), except for B(M,C)O, which is shown to assume 4 m^2^/g. Generally, substitution with Sr or Co is shown to increase the accessibility of the otherwise hardly accessible surfaces of pure mixed oxides.

**Table 2:** Specific surface area values (S_BET_) as determined for the test catalysts by N_2_ sorptiometry.

Catalyst:	BFO	(B,S)FO	LFO	L(F,C)O	BMO	B(M,C)O	LMO	(L,S)MO
S_BET_ / ± 1 m^2^/g	2	20	6	21	10	4	16	20

### Catalyst activity

In situ IR gas-phase spectra taken from a 30-Torr portion of 2-PrOH molecules, after a 5-min contact period elapsed with a given amount of test catalysts at RT and 250 °C, are shown in Supplementary Figs. S8–S10. Irrespective of the test catalyst, the RT-spectra monitored nothing but the diagnostic absorption bands of the alcohol, namely, νOH at 3640, νCH at 2975 and 2889, δ(CH_3_)_as_ at 1468, δ(CH_3_)_s_ at 1382, δOH at 1244, νCO at 1149, νCC at 1072 and 951/cm^30^. On the other hand, all of the 250 °C spectra obtained are overwhelmed by strong bands of the alcohol dehydrogenation product (acetone) molecules, i.e. νC=O at 1735, δCH at 1451 and 1382, νCC at 1218, (CH_3_)_r_ at 1072/cm^31^. The spectra monitored none of the diagnostic bands of the alcohol dehydration product (propene) molecules (at 1831, 1655, 988, 952, and 913/cm)^30^. This indicates that all of the test catalysts are almost 100% dehydrogenation selective. Accordingly, handling the integrated peak area of the alcohol νOH-band (at 3640/cm), as monitored in each of the 250 °C spectra versus that monitored in the RT-spectrum, resulted in calculating the alcohol conversion turn-over-numbers (TON/2-PrOH molecules converted/min m^2^-Catalyst) set out in Table 3.

**Table 3:** Turn-over-number (TON) of the dehydrogenation of gas-phase 2-PrOH molecules on the catalysts at 250 °C for 5 min as determined by in-situ IR spectroscopy.

Catalyst	LCI/+ve		TON/(10^19^ 2-PrOH molecules converted/min m^2^-Cat)	
	Formal	Relative^a^	Individual	Average^b^
BFO	0.0	0.0	3.00	2.84
LFO			2.72	
L(F,C)O			2.80	
LMO	0.3	0.10	3.15	3.15
(L,S)MO		0.30	3.80	3.80
(B,S)FO	0.6	0.20	3.54	3.54
BMO	1.5	0.83	6.04	6.04
B(M,C)O		0.66	5.04	5.04

^a^It is relative LCI and was calculated for biphasic catalysts considering the contribution of each XRD-identified phase to the formal LCI based on the phase's relative abundance (approximated from the relative intensity of the strongest XRD peak of each phase). ^b^The average TON values determined for various catalysts with the same LCI value = ∑^n^ TON/n, where n, number of TON values.

**Figure 1:**
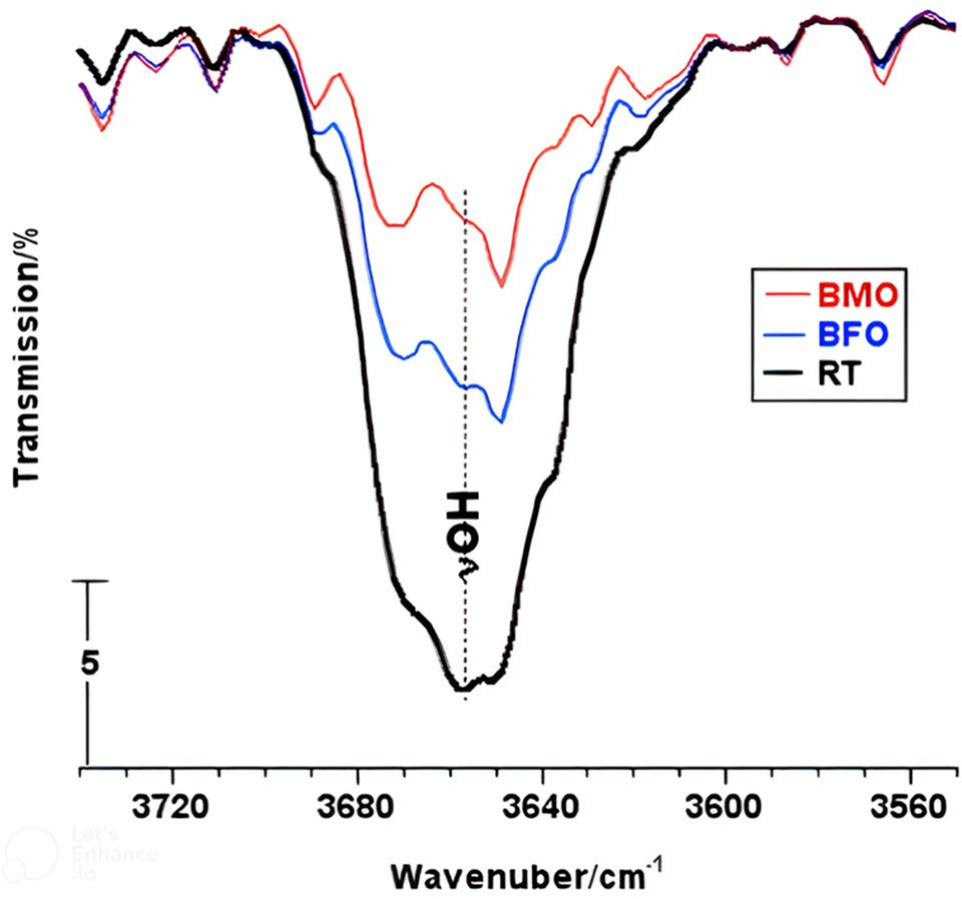
In situ IR spectra displaying peak area changes conceded by the 2-PrOH hydroxyl group absorption band (at RT) after a 5-min contact (at 250 °C) with the indicated test catalysts.

Figure 1 compares the alcohol-νOH peak area at RT and 250 °C after a 5-min contact period with the test catalysts BMO and BFO. Compatibly, Table 3 shows that BMO exhibits a TON twice that exhibited by the BFO catalyst (viz., 6.04 vs. 3.0 × 10^19^ 2-PrOH molecules converted/min m^2^-Cat). Furthermore, Table 3 shows that manganite-based pure and substituted mixed oxides have, in general, stronger 2-PrOH dehydrogenation activity than the ferrite-based catalysts tested.

### The activity-lattice charge relationship

**Figure 2:**
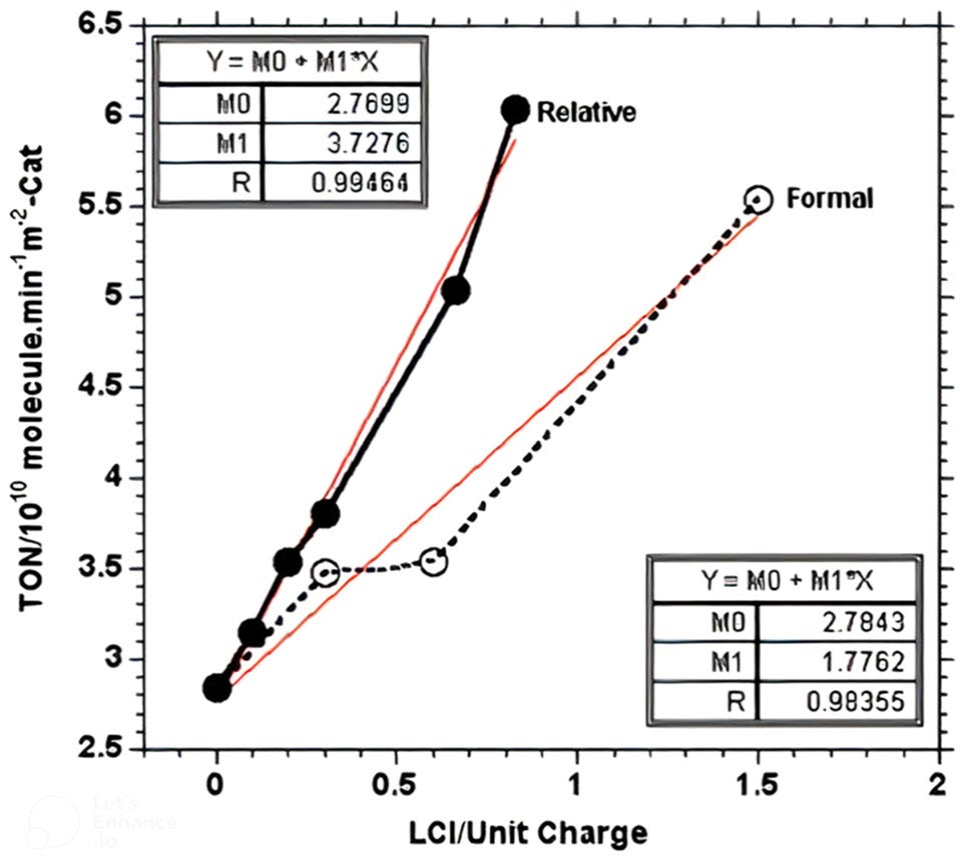
TON-LCI plot constructed using the average TON value determined on test catalysts exhibiting the same excess positive charge.

Table 1 reveals that the apparent distinction between the manganite- and ferrite-based catalysts lies in the charge imbalance of crystal lattices of the former set of catalysts, manifested in excess positive charge. The magnitude of the excess charge was found by applying molecular stoichiometry calculations to each catalyst's chemical composition of the XRD-identified phases. To determine whether a relationship exists between the alcohol dehydrogenation activity and the lattice-charge imbalance, the average value calculated for TON determined on test catalysts having the same excess charge is plotted as a function of the relative LCI value in Fig. 2. Relative LCI was calculated considering the relative abundance of the XRD-identified phases as approximated by the relative ratio of the diagnostic strongest diffraction peak of each phase. It is evident from Fig. 2 that the linearity of the TON value as a function of the relative LCI is better than that as a function of the formal LCI. The linear relationships obtained may assume that the surface excess charges are the surface attribute of the catalysts’ dehydrogenation activity, notably that the intrinsic nature of the TON values (per m^2^-Cat) excludes the surface area from playing any role in shaping this relationship. Nevertheless, the likely coexistence of other surface attributes cannot be excluded with certainty.

### Surface manifestation of the charge imbalance

The lattice-charge imbalance is shown (Tables 1 and 3) to be reflected solely in (a net) excess, positive charge. This result may imply the establishment on test catalysts of Zener phase-like species^15,28^. These species, which may be diagnosed by corresponding visible-light excited *d–d* electron double-exchange interactions (peaking at 498 and 555 nm; Table 1) have been suggested to form by bridging transition metal ions of different oxidation states via oxide ions, viz. M^n+^–O^2−^–M^(n+1)+^ species^28^. Table 1 shows that the formation of such species is more likely on manganite- than ferrite-based mixed oxides. Indeed, the oxidation of Mn^3+^ to higher valence states (Mn^>3+^) is much more feasible electronically and thermodynamically than Fe^3+^^6^. To determine the surface chemical manifestation of the lattice-charge imbalance (i.e. the excess positive charge), the pure and substituted, manganite-based mixed oxides were examined by XPS. The obtained full spectra (not shown) monitored photoelectron emissions from C1s, O1s, and Mn2p on surfaces of all of the catalysts examined, but from Co2p and/or Bi4f only on BMO and B(M,C)O, and Sr3d and/or La3d only on LMO and (L,S)MO. These results ensure uniform exposure of the bulk elemental composition on the surfaces of the test samples.

**Figure 3:**
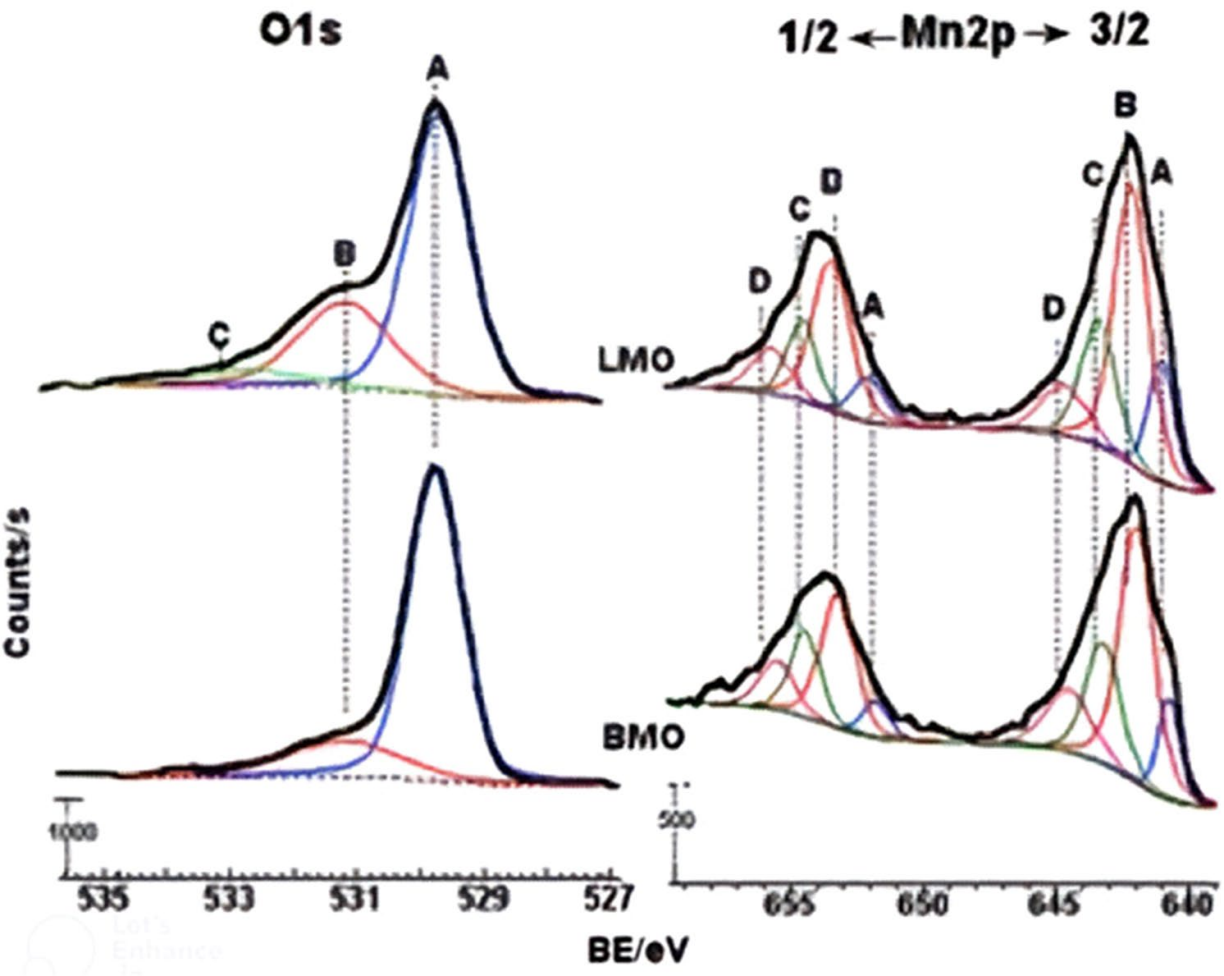
Deconvoluted XPS O1s and Mn2p spectra obtained for BMO and LMO [**A**–**D** label various oxidation states resolved for the two elements].

XPS spectra monitoring peaks of O1s, Mn2p, Bi4f, and La3d photoelectron emissions are presented, assigned and discussed, as a function of the test catalyst in Supplementary Figs. S11–S14 and the related text. O1s and Mn2p spectra obtained for BMO and LMO are compared, for example, in Fig. 3. The identification and quantification results derived for all of the elements encountered on the four test catalysts are presented in supplementary Table S2 (I–IV). On the other hand, relative proportions calculated from the atomic ratios (Supplementary Table S2) derived for the resolved oxygen and manganese species (in Fig. 3) are presented in Table 4.

**Table 4:** Relative atomic proportions calculated using the atomic ratios derived from the XPS-monitored O1s and Mn2p states shown in Supplementary Table S2.

Catalyst	(O_t_/M_t_)_obs_^a^	(O_t_/M_t_)_exp_^b^	O_v_/O_t_^c^	Mn^>3+^/Mn_t_	Mn^<3+^/Mn_t_
BMO	2.03	1.53–1.66	0.20	0.42	0.11
B(M,C)O	1.70	1.54–1.66	0.15	0.41	0.06
(L,S)MO	2.40	1.58	0.26	0.38	0.16
LMO	2.50	1.50–1.58	0.32	0.35	0.15

^a^XPS-determined total atomic percentage of catalyst surface oxygen (O_t_) and metal (M_t_) contents. ^b^As expected from the chemical and phase compositions of the catalysts. ^c^O_v_, oxygen adsorbed on vacant sites.

The downward order of the catalysts in the 1st column of Table 4 parallels the descending order of their 2-PrOH dehydrogenation activities (*cf.* TON values in Table 3). Correspondingly, Table 4 shows that the higher the proportion of the surface Mn^>3+^ sites (and the lower the proportions of the Mn^<3+^ and O_v_ sites), the higher the alcohol dehydrogenation activity. Table 4 shows, moreover, that the presence of La in the chemical makeup of the catalyst renders the observed O_t_/M_t_ ratio (2nd column) much higher than the expected ratio (3rd column). This latter relationship is strongly connected with the XPS- (O1s-C and C1s-C at 533.07 and 288.79 eV, respectively, Supplementary Table S2 (II & IV) and IR-observed (νCO_3_^2−^ at 1468 and 1060/cm, Supplementary Fig. S5) La-enhancement of the surface basicity and, hence, ambient-CO_2_ chemisorption on LMO and (L,S)MO.

The XPS results (Tables 4 and supplementary S2; Fig. 3 and supplementary S12) demonstrate the exposure on the manganite-based catalysts of manganese sites assuming a variety of > 3+ oxidation states, as well as manganese sites in the di-valent state. The visible-light absorption observed for these catalysts at 498 and 555 nm (Table 1) may account for the involvement of these various oxidation states of Mn in oxygen-mediated Zener-like linkages (Mn^n+^–O^2−^–Mn^(n+1)+^), which are known to facilitate via-oxygen *d–d* electron double-exchange interactions^28^. According to Nohman et al.^29^, Zener-like surface species (M^n+^–O^2−^–M^(n+1)+^) may provide coordination sites for the adsorption of reactant molecules and an electron-mobile environment for redox surface reactions. Compatibly, one may consider the following 2-PrOH dehydrogenation reaction mechanism^30,32^ to occur on the present test catalysts, particularly the manganite-based catalysts:

$$ \left( {{\text{CH}}_{{3}} } \right){\text{CHOH}}\left( {\text{g}} \right) \, + {\text{ M}}^{{\left( {{\text{n}} + {1}} \right) + }} \left( {\text{s}} \right) \, + {\text{ O}}^{{{2} - }} \left( {\text{s}} \right) \, \leftrightarrow \, \left( {{\text{CH}}_{{3}} } \right)_{{2}} {\text{CHO}}^{ - } {\text{M}}^{{\left( {{\text{n}} + {1}} \right) + }} \left( {\text{s}} \right) \, + {\text{ OH}}^{ - } \left( {\text{s}} \right) $$

$$ {\text{(CH}}_{{3}} )_{{2}} {\text{CHO}}^{ - } {\text{M}}^{{\left( {{\text{n}} + {1}} \right) + }} \left( {\text{s}} \right) \leftrightarrow \left( {{\text{CH}}_{{3}} } \right)_{{2}} {\text{C}} = {\text{O}}\left( {\text{g}} \right) \, + {\text{ HM}}^{{{\text{n}} + }} \left( {\text{s}} \right) $$

$$ {\text{HM}}^{{{\text{n}} + }} \left( {\text{s}} \right) \, + {\text{ OH}}^{ - } \left( {\text{s}} \right) \leftrightarrow {\text{H}}_{{2}} \left( {\text{g}} \right) \, + {\text{ O}}^{{{2} - }} \left( {\text{s}} \right) \, + {\text{ M}}^{{\left( {{\text{n}} + {1}} \right) + }} \left( {\text{s}} \right) $$

## Conclusion

The above-presented and discussed results may help draw the following conclusions:

1. Bi (versus La) facilitates the formation of lattice-charge imbalanced polymeric crystalline phases, particularly in manganite-based mixed oxides.
2. The lattice-charge imbalance is manifested at the surface in generating Mn sites assuming various oxidation states ≥ 3+.
3. Consequently, electron double-exchange-facilitating Mn^n+^–O^2−^–Mn^(n+1)+^ type linkages are formed, and hence, a redox catalytic activity-necessitated electron-mobile environment is established at the surface.
4. All of the present manganite- and ferrite-based mixed oxide catalysts, whether pure, Sr- or Co-substituted, are selective 2-propanol dehydrogenation catalysts.
5. Whether pure or Co-substituted, bismuth manganites are almost twice as active as the corresponding ferrite-based catalysts.
6. In general, the dehydrogenation activity is almost directly linearly related to the magnitude (i.e. the net positive charge) of the lattice-charge imbalance of the test catalysts.
7. Using more acidic counter ions (*A* = Bi *instead* of La) and more oxidizable transition metal ions (*B* = Mn *instead* of Fe) may improve the redox catalytic activity of perovskite-type mixed oxides.
8. The presence and magnitude of lattice-charge imbalance may help predict the redox activity of perovskite-type mixed oxide catalysts.

## Supplementary Information

Supplementary Information.

## Data Availability

The data that support the findings of this study are available from the following source databases: International Center for Diffraction Data (ICDD), https://www.ICDD.com/PDF-2--ICDD.com AND/OR. American Mineralogist Crystal Structure Database: http://rruff.geo.arizona.edu/AMS/amcsd.php. NIST XPS database: https://srdata.nist.gov/xps/. However, restrictions apply to the availability of the ICDD XRD standard data, which were used under license and are not publicly available. Therefore, the link to the publicly available alternative American Mineralogist Crystal Structure database is given. In the main window, the following options must be chosen: diffraction data and amc long form, before choosing “diffraction search”. In the consequent emerging window, the following choice must be made: 2-theta, before inserting the value of the intensity cut off (above 5%), and the radiation wavelength (1.5418 Angstrom). Then, the 2-theta values (and their tolerance) of the monitored peaks in the observed diffractogram must be entered in succession and then submitted. In the re-emerging main window, “Search” was chosen. Subsequently, the matching standard diffraction data will be reported. Nevertheless, the reference XRD data used in the present investigation (ICDD-sourced) are available from the authors upon reasonable request (*cf.* Prof. M.I. Zaki).
