# Supplementary material for: Lattice-charge imbalance and redox catalysis over perovskite-type ferrite- and manganite-based mixed oxides as studied by XRD, FTIR, UV–Vis DRS, and XPS
Source: Sci Rep. 2023 May 8;13:7453. doi: 10.1038/s41598-023-34065-3 (PMC10167207; doi:10.1038/s41598-023-34065-3)
Supplement: Supplementary file 1 — Supplementary Information. [file 41598_2023_34065_MOESM1_ESM.docx]

*Supplementary Information*

**Lattice-charge Imbalance and Redox Catalysis over Perovskite-type Ferrite- and Manganite-based Mixed Oxides as studied by XRD, FTIR, UV-Vis DRS, and XPS**

Gamal A.H. Mekhemer,^1^ Hagar A.A. Mohamed,^1^ Ali Bumajdad,^2^ Mohamed I. Zaki^1,*^

*^1^Chemistry Department, Faculty of Science, Minia University, El-Minia 61519, Egypt.*

*^2^Chemistry Department, Faculty of Science, Kuwait University, P.O. Box 5969 Safat, 13060 Kuwait.*

**^*^ Corresponding author**

**Mohamed I. Zaki**

**Professor, Chemistry Department**

**Faculty of Science,**

**Minia University,**

**El-Minia 61519,**

**EGYPT**

E-mail: [mizaki@mu.edu.eg](mailto:mizaki@mu.edu.eg)

Tel.: 00201202149149

**Results and discussion**

**Catalyst characteristics**


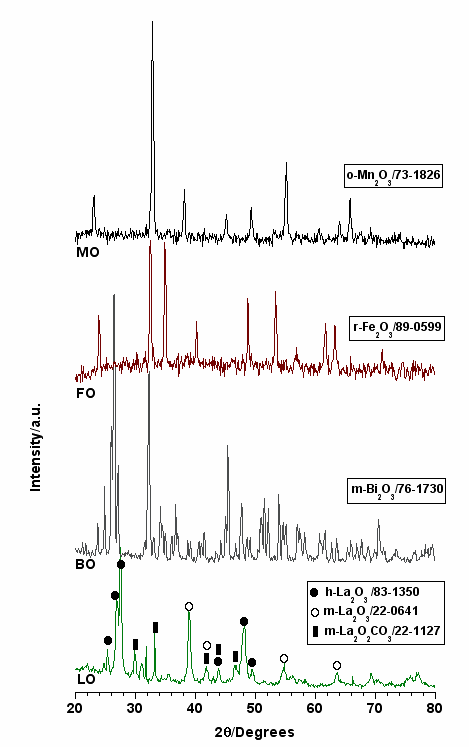


**Figure S1.** XRD powder diffractograms obtained for the indicated simple oxides

**Figure S1** stacks XRD powder diffractograms obtained for the simple oxides MO, FO, BO, and LO. Consulting the standard JCPDS data files^1^ specified correspondingly in the figure, crystalline domains in MO, FO or BO are found to be made up of a single-phase, which is orthrhomic(o)-Mn_2_O_3_, rhombohedral(r)-Fe_2_O_3_, or monoclinic(m)-Bi_2_O_3_, respectively. In contrast, LO is found to be multi-phasic, since it is majored by hexagonal(h)-La_2_O_3_, and minored by m-La_2_O_3_ and m-La_2_O_2_CO_3_. Most likely, it is the known strong basicity of lanthanum oxide that helped develop a strong tendency towards adsorption/absorption of ambient CO_2_ molecules, which lead to the formation of the minority oxy-carbonate phase. These results indicate that the metal-oxygen species contained in MO, FO, BO, and LO are favorably ordered into the identified, thermodynamically stable simple oxides formed at 700 ^o^C once not engaged in competing interaction(s) with other metal ions or components of the ambient atmosphere.


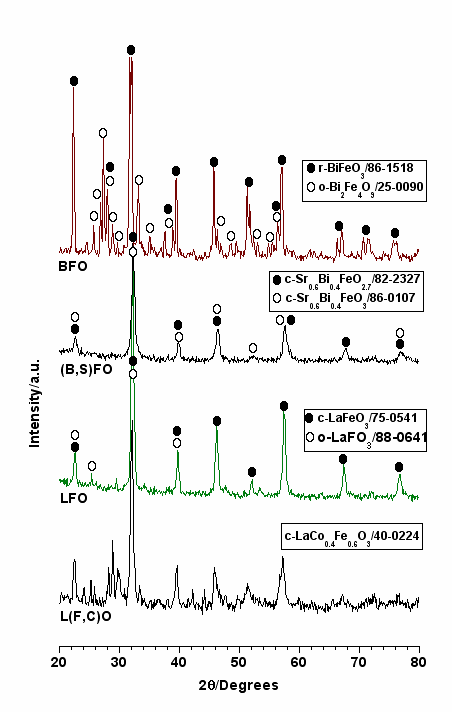


**Figure S2.** XRD powder diffractograms obtained for the indicated ferrite-based, pure and substituted mixed oxides.

**Figure S2** compares XRD diffractograms obtained for the 700 ^o^C calcination products of the indicated ferrite-based, pure and substituted mixed oxides. Matching the monitored diffraction patterns with the indicated JCPDS standard data^1^ reveals that the crystalline bulk structure of pure BFO is majored by r-BiFeO_3_ and minored by polymeric o-Bi_2_Fe_4_O_9_ phase. Analogous behavior is shown for pure LFO, which is majored by cubic(c-)LaFeO_3_ and minored by o-LaFeO_3_. When one-fifth of the initial content of Bi in BFO was replaced by Sr, the crystalline structure of the resulting (B,S)FO is shown to be majored by the oxygen-deficient ternary-metal c-Sr_0.6_B_0.4_FeO_2.7_ phase and minored by its stoichiometric modification, i.e. the ternary-metal c-Sr_0.6_Bi_0.4_FeO_3_ phase. On the other hand, a similar one-fifth substitution of the Fe content of LFO by Co is shown to give rise to a sole crystalline phase made up by the ternary-metal c-LaCo_0.4_Fe_0.6_O_3_ phase. Accordingly, XRD-results displayed in **Fig. S2** may help infer that the crystalline bulk structure of 700-^o^C calcined pure and substituted ferrite-based mixed oxides is composed of perfect (cubic) and distorted (to orthorhombic or rhombohedral) perovskite-type phases, only one of them suffers a formal lattice-charge imbalance (namely, the c-Sr_0.6_Bi_0.4_FeO_3_ with +0.6 excess charge). BFO is shown to allow for the formation of a crystalline polymeric phase (o-Bi_2_Fe_4_O_9_), whereas LFO is shown not to do likewise. Neither of the crystalline bulk structures formed facilitates the segregation of XRD-detectable simple oxide phases.


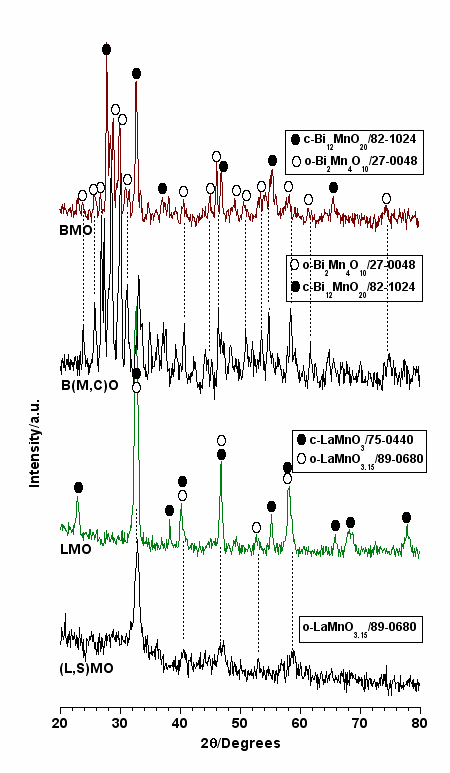


**Figure S3.** XRD powder diffractograms obtained for the indicated manganite-based, pure and substituted mixed oxides.

XRD results communicated in **Fig. S3** for the 700 ^o^C calcination products of pure and substituted manganite-based mixed oxides show the Bi, in the presence of Mn, to encourage the formation of polymeric mixed oxides (namely, o-Bi_2_Mn_4­_O_10_ and c-Bi_12_MnO_20_) more than in the corresponding ferrite-based mixed oxides (**Fig. S2**). Whereas La remains supporting the formation of non-polymeric perovskite-type oxide phases. Neither of the substituted manganite-based oxides is shown to form ternary-metal phases. However, all of the manganite-based mixed oxides are shown to give rise to crystalline phases assuming charge-imbalanced lattices. Hence, irrespective of the counter ion (Bi or La) the presence of Mn (*versus* Fe) allows for the formation of mixed oxides organized in charge-imbalanced lattices and does not facilitate the formation of ternary-metal crystalline phases. On the other hand, the presence of Bi (*versus* La) facilities the formation of polymeric crystalline phases particularly in the presence of Mn.


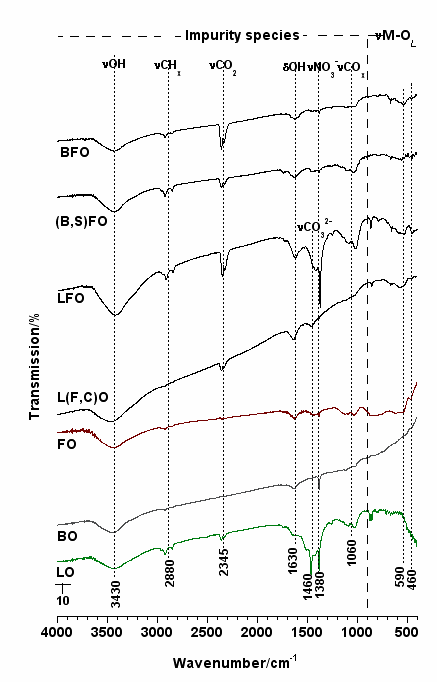


**Figure S4.** *Ex-situ* IR spectra obtained for the indicated ferrite-based, pure and substituted mixed oxides. The spectra obtained for the indicated simple oxide are included for comparison purposes.


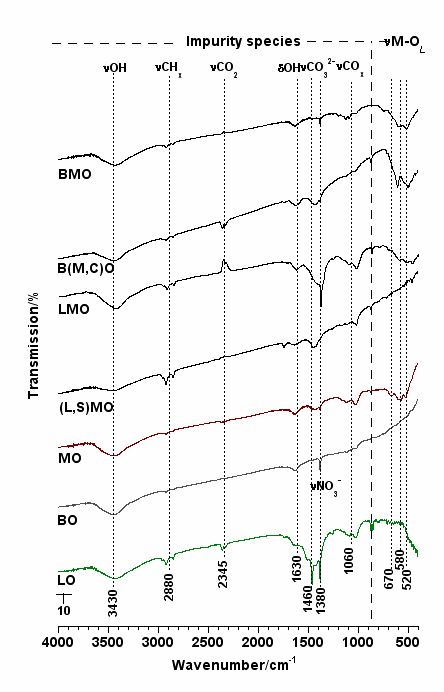


**Figure S5.** *Ex-situ* IR spectra obtained for the indicated manganite-based, pure and substituted mixed oxides.

**Figures S4 and S5** compare *ex-situ* IR spectra taken, respectively, from ferrite- and manganite-based sets of mixed and simple metal oxides. The spectra discern two frequency ranges of which the higher frequency range (>1000 cm^-1^ ) is shown to monitor absorption bands due to impurity species, whereas the lower one (<1000) resolves bands due to the metal-oxygen lattice vibrations^2^ . The high-frequency bands are attributable to the presence of surface hydroxyl (νOH at 3430 cm^-1^ ), water (δOH at 1630 cm^-1^), hydrocarbon (νCH, bands centered around 2880 cm^-1^ ), nitrate (νNO_3_^-^ at 1380 cm^-1^), and carbonate (νCO_3_^2-^ at 1468 and 1060 cm^-1^) impurity surface species^3^. The band resolved at 2345 cm^-1^ is due to a miss-cancelation of νCO_2_ absorption of ambient CO_2_ molecules. Considering the spectra taken from the simple oxides (LO, BO, FO, and MO; **Figs. S4** and **S5**), one can easily relate the carbonate bands (at 1460 and 1060 cm^-1^) monitored in the spectrum of LO to its bulk minority phase of La_2_O_2_CO_3_ (**Fig. S1**). Hence, the surface carbonate species only observed on the pure LFO and LMO are associated with their La contents. Whereas, the absence of carbonate species on L(F,C)O and (L,S)MO may imply that the substituent ions (Sr or Co) render the La content either inaccessible or incapable of chemisorbing ambient CO_2_ molecules.


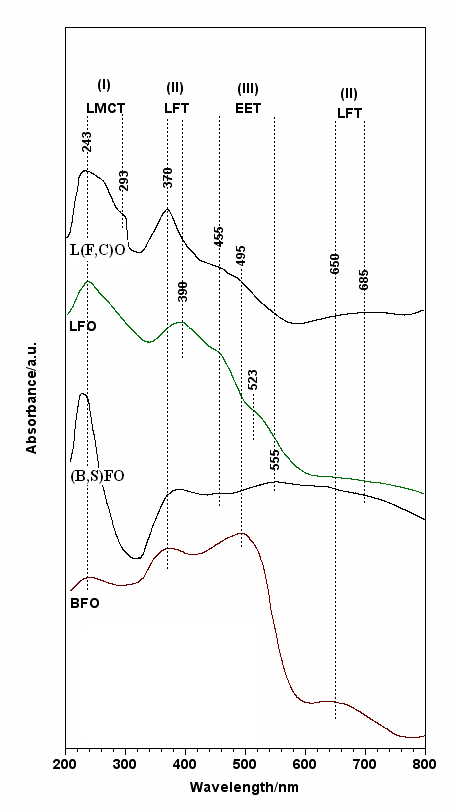


**Figure S6.** UV-Vis DRS spectra obtained for the indicated ferrite-based, pure, and substituted mixed oxides [LMCT = ligand-metal charge transfer transitions, LFT = ligand field transitions, EET = electron exchange transitions].


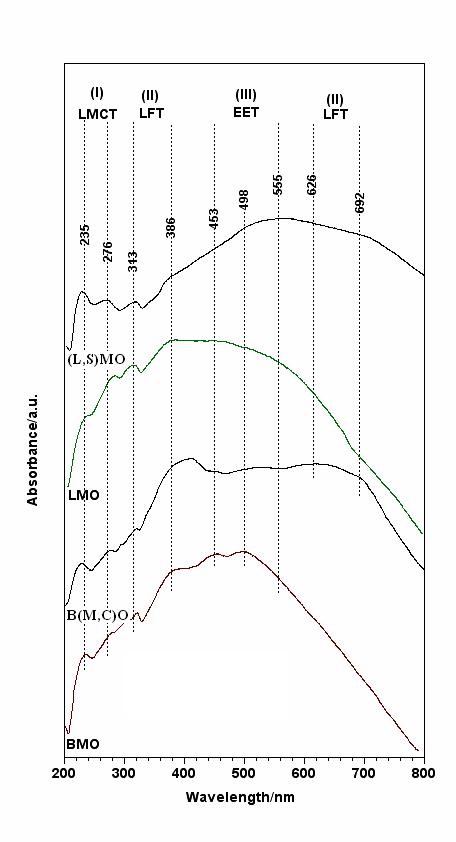


**Figure S7.** UV-Vis DRS spectra obtained for the indicated manganite-based, pure, and substituted mixed oxides.

UV-Vis DRS spectra obtained for the ferrite-based test samples (**Fig. S6**) monitor mostly a single absorption (near 243 nm) in the UV region (≤300 nm), due to ligand-to-metal charge transfer (O^2-^→Fe^3+^ LMCT) transition, and two weak absorptions (at 370-390 and 650-685 nm) in the visible region (>300 nm), due to ligand-field transitions (Fe^3+^(d^5^)-Fe^3+^(d^5^) LFT). Additional absorptions monitored in the visible region (at 455-555 nm) are assignable to via-oxygen d-d electron exchange interactions (Fe^3+^-O^2—^Fe^3+^ EET)^4^. Analogous LMCT (at 235 and 276 nm), LFT (at 313, 386, 626, and 692 nm), and EET (at 453-555 nm) absorptions are monitored in the spectra obtained for the manganite-based samples (**Fig. S7**). However, the latter spectra are distinct by much stronger, and more extended EET and LFTabsorptions than those monitored in the spectra obtained for the ferrite-based samples (**Fig. S6**). This may imply that the manganite-based samples enjoy higher contents of associated Mn-O-Mn species involving Mn cations not only of like oxidation states (3+) but also of dislike oxidation states (≥3+)^4^.

**Catalyst activity**


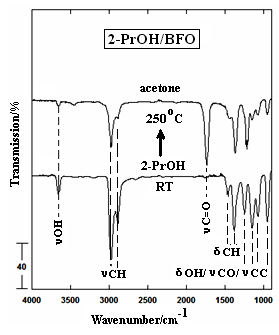


**Figure S8.** *In-situ* FTIR transmission spectra taken from 10-Torr 2-PrOH vapor over the indicated ferrite-based catalyst, following a 5-min contact at RT and 250 ^o^C.

**Figure S8** compares *in-situ* FT-IR gas-phase spectra taken from 10-Torr 2-PrOH gas phase after a 5-min contact with BFO catalyst at two different reaction temperatures: room temperature (RT) and 250 ^o^C. The RT-spectrum monitors, exclusively, diagnostic absorption bands of 2-PrOH: νOH at 3640, νCH at 2975 and 2889, δ(CH_3_)_as_ at 1468, δ(CH_3_)_s_ at 1382, δOH at 1244, νCO at 1149, νCC at 1072 and 951cm^-1 5^. The analytical band of these is the νOH at 3640 cm^-1^, whose integrated area has, frequently, been used to account for the amount of the alcohol molecules^6^. On the other hand, the 250-^o^C spectrum is shown to be relieved considerably of most of the alcohol diagnostic absorptions, monitoring instead diagnostic absorptions of its dehydrogenation product; i.e., acetone molecules: νC=O at 1735, δCH at 1451, and 1382, νCC at 1218, (CH_3_)_r_ at 1072 cm^-1 7^. It is worth noting, that none of the diagnostic absorption bands (at 1831, 1655, 988, 952, and 913 cm^-1 5^) of the alcohol dehydration product (propene molecules) is observed in the latter spectrum.


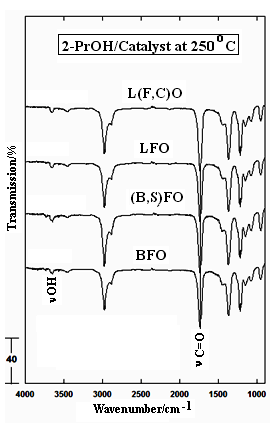


**Figure S9.** *In-situ* FTIR transmission spectra taken of 10-Torr 2-PrOH vapor over the indicated ferrite-based catalysts, following a 5-min contact at 250 ^o^C.


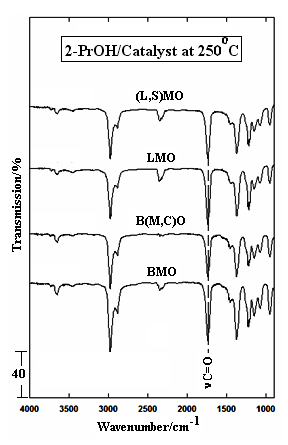


**Figure S10.** *In-situ* FTIR transmission spectra taken of 10-Torr 2-PrOH vapor over the indicated manganite-based catalysts, following a 5-min contact at 250 ^o^C.

**Figures S9 and S10** compare the 250-^o^C gas-phase spectra obtained, respectively, over the indicated ferrite- and manganite-based test catalysts, which are shown to be overwhelmed by strong diagnostic absorptions of acetone molecules. Though weak diagnostic absorptions of unconverted 2-PrOH molecules may, still, be resolved (particularly the νOH absorption at 3640 cm^-1^), none of the diagnostic absorptions of propene molecules is observable in the spectra. These spectra differ amongst themselves in the intensity of the νOH absorption of the unconverted alcohol molecules.

**Surface manifestation of the charge imbalance**


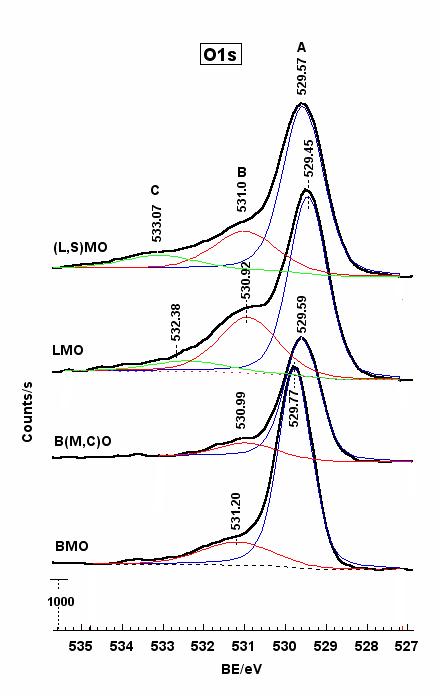


**Figure S11.** Deconvoluted XPS O1s spectra obtained for the indicated manganite-based catalysts [A-C denote various oxygen-containing species].

XPS O1s spectra compared in **Fig. S11** are similar in resolving a strong peak (A) at 529.77-529.45 eV and a weak peak (B) at 531.20-530-92 eV. A third, and a still weaker peak (C) is resolved at 533.07-532.38 eV only for the La-containing manganite-based catalysts, viz. LMO and (L,S)MO catalysts. The strong peak-A assumes binding energy (BE) values in the range (590-580 eV) frequently assigned for lattice-oxide (denoted O_L_) species of metal oxides^8-10^. Therefore, the oxygen involved is bound indiscriminately to the metal constitution of the mixed oxide catalyst (and described as O-metal). This may explain its relatively highest BE value (529.77 eV) when bound to the acidic Bi/Mn in BMO, and its lowest BE value (529.45 eV) when bound to the basic La/Mn in LMO. On the other hand, the weak peak-B assumes BE values in the range 530-531 eV, which is occasionally used to characterize oxygen bound to vacancy sites (described as O-vacancy and denoted O_V_)^9,10^. These oxygen species are usually charged molecular (O_2_^x-^) or atomic species (O^-^/OH^-^). The weakest third peak-C is observed in the high BE range 533.07-532.38 eV attributed to oxygen in metal-carbonate species (i.e. M-O-CO_2_ or M-O-C(O)-O-M)^8^. The fact that it is monitored only in the spectra obtained for LMO and (L,S)MO may attribute the carbonate formation to the La-enhanced adsorption of ambient CO_2_ molecules. It is worth mentioning, that carbonate species are recognizable in the IR spectra of LMO and (L,S)MO in **Fig. S5**.


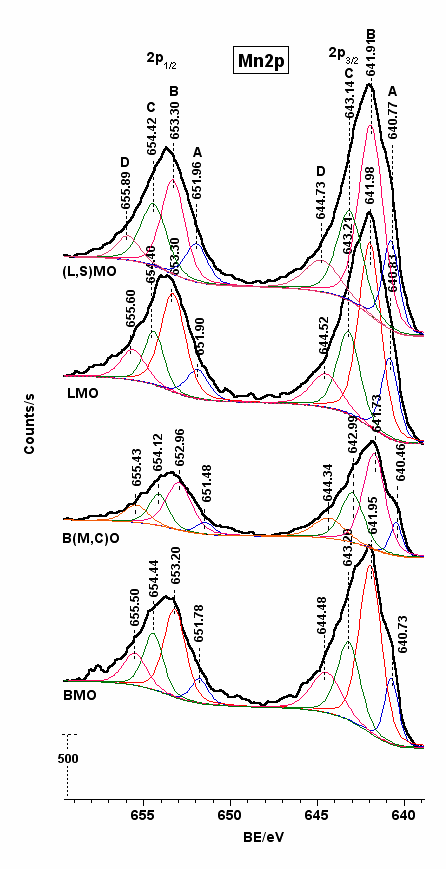


**Figure S12.** Deconvoluted XPS Mn2p spectra obtained for the indicated manganese-based catalysts [A-D denote Mn in various oxidation states].

XPS Mn2P spectra exhibited in **Fig. S12** are similar in resolving 4 peaks both in the spin-orbit doublet 2p_3/2_ and 2p_1/2_ BE ranges (645-640 and 656-651 eV, respectively) [8,11]. According to Zaki and Kappenstein^11^, the 4 peaks account for Mn having various oxidation states Mn^<3+^ (peaks-A), Mn^3+^ (peaks-B), and Mn^>3+^ (peaks-C and –D) (**Fig. S12**). The following **Table S1** compares the observed and reported XPS Mn2p peaks for various oxidation states of Mn in simple and composite Mn-oxides. It is obvious from **Table S1** that Mn sites exposed on the test catalysts assume a wide range of oxidation states (from 2+ to ≥3+). The presence of the di-valent state may help presuming that initial Mn^3+^ may have undergone a disproportionation-type of reaction : 2Mn^3+^ = Mn^2+^ + Mn^4+^  ^11^.

| **Observed BE/eV** | | | **Reported BE/eV** | | | **Oxidation**  **state** | **Source**  **Reference** |
| --- | --- | --- | --- | --- | --- | --- | --- |
| **Peak** | **Mn2p_3/2_** | **Mn2p_1/2_** | **Compound** | **Mn2p_3/2_** | **Mn2p_1/2_** |  |  |
| **A** | 640.46 –  640.83 | 651.48 –  651.96 | MnO,  MnFe_2_O_4_ | 640.40 –  640.80 | 651.50 –  653.40 | Mn^2+^ | [8] |
| **B** | 641.73 –  641.98 | 652.96 –  653.30 | Mn_2_O_3_,  Mn_3_O_4_ | 641.1 –  642.0 | 652.70-  653.70 | Mn^3+^ a/o^a^  M^3+^/Mn^2+^ | [8,11] |
| **C** | 642.99 –  643.21 | 654.12 –  654.44 | MnO_2_,  Mn_5_O_8_ | 641.60 –  643.40 | 653.81 –  654.12 | Mn^4+^ a/o  Mn^4+^/Mn^2+^ | [8,11] |
| **D** | 644.34 –  644.73 | 655.43 –  655.89 | KMn_8_O_16_,  KMnO_4_ | 643.4 –  647.0 | 653.70 –  654.80 | Mn^6+^ a/o  Mn^4+^/Mn^6+^ | [8] |

**Table S1.** Observed (Fig. S12) and reported BE values for XPS Mn2p photoelectron emissions. ^a^ a/o = and/or.

**
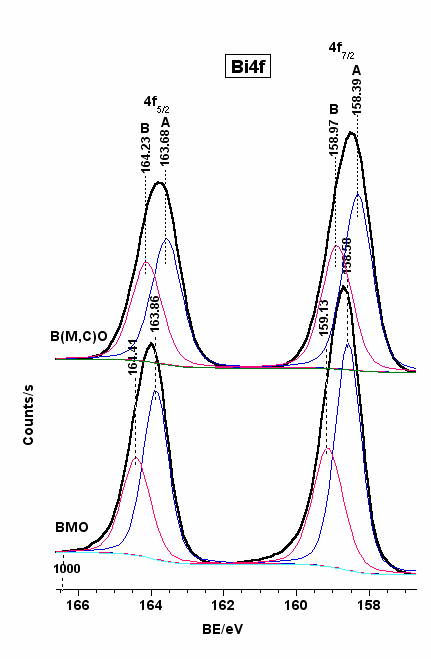
**

**Figure S13.** Deconvoluted XPS Bi4f spectra obtained for the indicated manganese-based catalysts [A and B denote two different states of Bi].

XPS Bi4f spectra obtained for BMO and B(M,C)O are compared in **Fig. S13**. The two spectra are similar in monitoring the spin-orbit doublet 4f_7/2_ and 4f_5/2_ ^10,12^. The deconvolution resolved two peaks (A and B) in each spin-orbit state: A, 4f_7/2_ at 158.39 and 4f_5/2_ at 163.68 eV; and B, 4f_7/2_ at 158.97 and 4f_5/2_ at 164.23 eV. Accordingly, the spin-orbit splitting energy of the Bi4f doublet lies at 5.26 and 5.31 eV for states A and B, respectively, which are rather close to the expected value (5.31 eV) of Bi^3+^ ^9^. Thus, they may well be considered as accounting for the presence of Bi in the tri-valent state but in two different bonding modes/configurations. This outcome is quite compatible with the bi-phasic nature of the examined two catalysts BMO and B(M,C)O, where each phase assumes a different degree of polymerization (Bi_2_Mn_4_O_10_ and B_12_MnO_20_) and different extent of abundance in each catalyst (see Table 1).


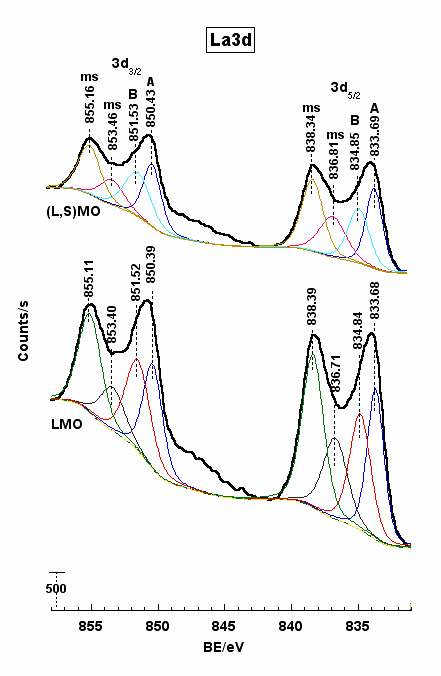


**Figure S14.** Deconvoluted XPS La3d spectra obtained for the indicated manganese-based catalysts [A and B denote two different states of La].

XPS La3d spectra obtained for LMO and (L,S)MO catalysts and compared in **Fig. S14** are similar in resolving two La3d spin-orbit doublets: A (3d_5/2_ at 833.69 and 3d_3/2_ at 850.43 eV) and B (3d_5/2_ at 834.85 and 3d_3/2_ at 851.53 eV). Moreover, they similarly resolve two different states of multiple splitting (ms). Recalling that the phase and chemical compositions of the two test catalysts (see Table 1) are closely similar (viz. LaMnO_3_ and LaMnO_3.15_), one may ascribe the two La states (A and B) to La^3+^ engaged in two different bonding systems. The reference data made available in NIST^8^ may help attribute state-A (3d_5/2_ at 833.69 and 3d_3/2_ at 850.43 eV) to La^3+^-O bonds similar to those in La_2_O_3_, whereas state-B (3d_5/2_ at 834.85 and 3d_3/2_ at 851.53 eV) to La^3+^-OCO_2_ bonds similar to those in La_2_(CO_3_)_2_ or La_2_O_2_(CO_3_).

**I: BMO surfaces**

| **Name** | **Peak BE** | **FWHM eV** | **Area (P) CPS eV** | | **Atomic %** |
| --- | --- | --- | --- | --- | --- |
| C1s A | 284.63 | 1.09 | 605.61 | 14.51 | |
| C1s B | 285.30 | 1.31 | 240.36 | 5.76 | |
| O1s A | 529.77 | 0.97 | 4477.97 | 42.99 | |
| O1s B | 531.20 | 2.09 | 1088.18 | 10.45 | |
| Mn2p, 3/2 A | 640.73 | 0.95 | 579.98 | 0.95 | |
| Mn2p, 1/2 A | 651.78 | 1.17 | 265.49 | 0.44 | |
| Mn2p, 3/2 B | 641.95 | 1.52 | 2330.85 | 3.83 | |
| Mn2p, 1/2 B | 653.20 | 1.49 | 1203.46 | 1.98 | |
| Mn2p, 3/2 C | 643.20 | 1.55 | 1116.97 | 1.83 | |
| Mn2p, 1/2 C | 654.44 | 1.33 | 687.87 | 1.13 | |
| Mn2p, 3/2 D | 644.48 | 2.06 | 759.14 | 1.25 | |
| Mn2p, 1/2 D | 655.50 | 1.58 | 462.25 | 0.76 | |
| Bi4f, 7/2 A | 158.58 | 0.84 | 5524.35 | 4.98 | |
| Bi4f, 5/2 A | 163.86 | 0.83 | 4093.02 | 3.69 | |
| Bi4f, 7/2 B | 159.13 | 1.00 | 3425.11 | 3.09 | |
| Bi4f, 5/2 B | 164.41 | 0.92 | 2615.25 | 2.36 | |

**II: LMO surfaces.**

| **Name** | **Peak BE** | **FWHM eV** | **Area (P) CPS.eV** | **Atomic %** |
| --- | --- | --- | --- | --- |
| C1s A | 284.66 | 1.20 | 857.83 | 16.16 |
| C1s B | 285.68 | 1.84 | 211.02 | 3.98 |
| C1s C | 288.79 | 1.40 | 142.35 | 2.68 |
| O1s A | 529.45 | 1.11 | 4600.56 | 34.73 |
| O1s B | 530.92 | 1.72 | 2185.86 | 16.51 |
| O1s C | 532.38 | 2.16 | 514.26 | 3.89 |
| Mn2p, 3/2 A | 640.83 | 1.17 | 903.62 | 1.17 |
| Mn2p, 1/2 A | 651. 90 | 1.51 | 425.87 | 0.55 |
| Mn2p, 3/2 B | 641.98 | 1.52 | 2694.34 | 3.48 |
| Mn2p, 1/2 B | 653.30 | 1.69 | 1593.62 | 2.07 |
| Mn2p, 3/2 C | 643.21 | 1.55 | 1240.90 | 1.60 |
| Mn2p, 1/2 C | 654.40 | 1.33 | 685.70 | 0.89 |
| Mn2p, 3/2 D | 644.52 | 2.06 | 666.26 | 0.86 |
| Mn2p, 1/2 D | 655.60 | 1.71 | 495.91 | 0.64 |
| La3d, 5/2 A | 833.68 | 1.49 | 3403.91 | 1.51 |
| La3d, 3/2 A | 850.39 | 1.71 | 2771.04 | 1.24 |
| La3d, 5/2 A Multiplet split | 836.71 | 2.15 | 2663.61 | 1.18 |
| La3d, 3/2 A Multiplet split | 853.40 | 2.17 | 1452.66 | 0.65 |
| La3d,5/2 B | 834.84 | 1.88 | 3313.11 | 1.47 |
| La3d, 3/2 B | 851.52 | 2.17 | 3023.79 | 1.36 |
| La3d, 5/2 B Mutiplet split | 838.39 | 1.91 | 4496.97 | 2.00 |
| La3d, 3/2 B Multiplet split | 855.11 | 2.01 | 3050.19 | 1.37 |

**III: B(M,C)O surfaces.**

| **Name** | **Peak BE** | **FWHM eV** | **Area (P) CPS.eV** | | **Atomic %** |
| --- | --- | --- | --- | --- | --- |
| C1s A | 284.62 | 1.03 | 407.32 | 15.03 | |
| C1s B | 285.20 | 0.89 | 157.01 | 5.80 | |
| O1s A | 529.59 | 1.02 | 2863.60 | 42.36 | |
| O1s B | 530.99 | 1.60 | 504.26 | 7.46 | |
| Mn2p, 3/2 A | 640.46 | 0.82 | 227.52 | 0.58 | |
| Mn2p, 1/2 A | 651.48 | 1.17 | 119.05 | 0.30 | |
| Mn2p, 3/2 B | 641.73 | 1.52 | 1218.30 | 3.08 | |
| M2p, 1/2 B | 652.96 | 1.76 | 691.95 | 1.76 | |
| Mn2p, 3/2 C | 642.99 | 1.55 | 641.52 | 1.62 | |
| Mn2p, 1/2 C | 654.12 | 1.33 | 348.91 | 0.89 | |
| Mn2p, 3/2 D | 644.34 | 2.06 | 343.77 | 0.87 | |
| Mn2p, 1/2 D | 655.43 | 1.58 | 227.86 | 0.58 | |
| Bi4f, 7/2 A | 158.39 | 0.94 | 4379.39 | 6.08 | |
| Bi4f, 5/2 A | 163.68 | 0.97 | 3257.68 | 4.53 | |
| Bi4f, 7/2 B | 158.97 | 0.99 | 3229.71 | 4.49 | |
| Bi4f, 5/2 B | 164.23 | 0.99 | 2613.50 | 3.63 | |
| Co2p A | 780.37 | 2.05 | 465.74 | 0.94 | |

**IV: (L,S)MO surfaces.**

| **Name** | **Peak BE** | **FWHM eV** | **Area (P) CPS.eV** | **Atomic %** |
| --- | --- | --- | --- | --- |
| C1s A | 284.69 | 1.18 | 1022.82 | 20.38 |
| C1s B | 285.91 | 1.35 | 77.35 | 1.54 |
| C1s C | 288.46 | 1.19 | 34.83 | 0.69 |
| O1s A | 529.57 | 1.18 | 4570.28 | 36.49 |
| O1s B | 531.00 | 1.82 | 1617.40 | 12.92 |
| O1s C | 533.07 | 2.14 | 652.88 | 5.22 |
| Mn2p, 3/2 A | 640.77 | 1.12 | 972.14 | 1.33 |
| Mn2p, 1/2 A | 651.96 | 1.51 | 562.97 | 0.77 |
| Mn2p, 3/2 B | 641.91 | 1.68 | 3096.81 | 4.23 |
| Mn2p, 1/2 B | 653.30 | 1.60 | 1452.63 | 1.99 |
| Mn2p, 3/2 C | 643.14 | 1.77 | 1537.99 | 2.10 |
| Mn2p, 1/2 C | 654.42 | 1.77 | 1055.96 | 1.45 |
| Mn2p, 3/2 D | 644.73 | 2.38 | 727.88 | 1.00 |
| M2p, 1/2 D | 655.89 | 1.58 | 368.91 | 0.51 |
| La3d, 5/2 A | 833.69 | 1.49 | 1801.40 | 0.85 |
| La3d, 3/2 A | 850.43 | 1.62 | 1565.82 | 0.74 |
| La3d, 5/2 A Multiplet split | 836.81 | 2.37 | 1472.57 | 0.69 |
| La3d, 3/2 A Multiplet split | 853.46 | 2.17 | 846.85 | 0.40 |
| La3d, 5/2 B | 834.85 | 1.88 | 1560.24 | 0.73 |
| La3d, 3/2 B | 851.53 | 2.32 | 1569.76 | 0.74 |
| La3d, 5/2 B Multiplet split | 838.34 | 1.91 | 2102.90 | 0.99 |
| La3d, 3/2 B Multiplet split | 855.16 | 2.01 | 1525.62 | 0.72 |
| Sr3d, 5/2 A | 132.25 | 1.01 | 131.11 | 0.49 |
| Sr3d, 3/2 A | 133.99 | 1.20 | 92.82 | 0.35 |
| Sr3d, 5/2 B | 133.16 | 1.37 | 235.68 | 0.88 |
| Sr3d, 3/2 B | 134.98 | 1.42 | 145.55 | 0.54 |
| Sr3d, 5/2 C | 134.64 | 1.74 | 206.99 | 0.77 |
| Sr3d, 3/2 C | 136.39 | 1.75 | 128.62 | 0.48 |

**Table S2.** XPS identification and quantification of elements observed on the examined manganite-based catalysts.

It is worth noting, that the C1s peak-C (288.79-288.46 eV) and O1s peak-C ( 533.07-532.38 eV) observed in the XPS data derived and tabulated (**Tables S2**) for the lanthanum-containing catalysts, namely LMO and (L,S)MO, are closely related to the IR-observed La-carbonate and oxy-carbonate species (**Fig. S5**).

**References**

1. International Center for Diffraction Data, Newton Square, PA 19073-13273.
2. Gadsden, J.A. Infrared Spectra of Minerals and Related Inorganic Compounds (Butterworths & Co, London, 1975).
3. Degen, I.A.Tables of Characteristic Group Frequencies for the Interpretation of Infrared and Raman spectra (Acolyte Pub, Harrow, 1997).
4. Alsalka, Y. *et al.* L.I. Iron-based photocatalytic and photo-electrocatalytic nano-structures: Facts, perspectives, and expectations., Appl. Catal. **244**, 1065-1095 (2019).
5. Hussein, G.A.M., Sheppard, N., Zaki, M.I. & Fahim, R.B. Infrared spectroscopic studies of the reactions of alcohols over group IVB metal oxide catalysts. Part-I: Propan-2-Ol over TiO_2_, ZrO_2,_ and HfO_2_, JCS Faraday Trans. **I 85**, 1723-1742 (1989).
6. Zaki, M.I., Mekhemer, G.A.H., Fouad, N.E. &. Rabee, A.I.M. Structure-acidity correlation of supported tungsten(VI)-oxo-species: FT-IR and TPD studies of adsorbed pyridine and catalytic decomposition of 2-propanol, Appl. Surf. Sci. **308**, 380-387 (2014).
7. Zaki, M.I., Hasan, M.A., Al-Sagheer, F.A. & Pasupulety, L. Surface chemistry of acetone on metal oxides: IR observation of acetone adsorption and consequent surface reactions on silica-alumina versus silica and alumina, Langmuir **16**, 430-436 (2000).
8. [WWW.NIST.com/XPS](http://WWW.NIST.com/XPS)
9. Ramadan, W. *et al.* Changes in the solid-state properties of bismuth iron oxide during the photocatalytic reformation of formic acid, Catal. Today **326,** 22-29 (2019).
10. Di, L., Yang, H., Xian, T. & Chen, X. Enhanced photocatalytic activity of NaBH_4_ reduced BiFeO_3_ nanoparticles for rhodamine B decolorization, Materials **10**, 1118-1129 (2017).
11. Zaki, M.I. & Kappenstein, C. X-ray photoelectron spectroscopy and diffractometry of MnO_x_ catalysts: Surface to bulk composition relationships, Z . Phys. Chem. (NF) **176**, 97-116 (1992).
12. Zaki, M.I., Ramadan, W., Katrib, A. & Rabee, A.I.M. Surface chemical and photocatalytic consequences of Ca-doping of BiFeO_3_ as probed by XPS and H_2_O_2_ decomposition studies, Appl. Surf . Sci. **317**, 929-943 (2014).
